# Tailoring combination HIV prevention for female sex workers

**DOI:** 10.1016/S2352-3018(18)30136-X

**Published:** 2018-07-18

**Authors:** Katrina F Ortblad, Catherine E Oldenburg

**Affiliations:** Department of Global Health and Population, Harvard T H Chan School of Public Health, Boston, MA, USA;; Francis I Proctor Foundation and Department of Ophthalmology, University of California, San Francisco, CA 94143, USA.

## Abstract

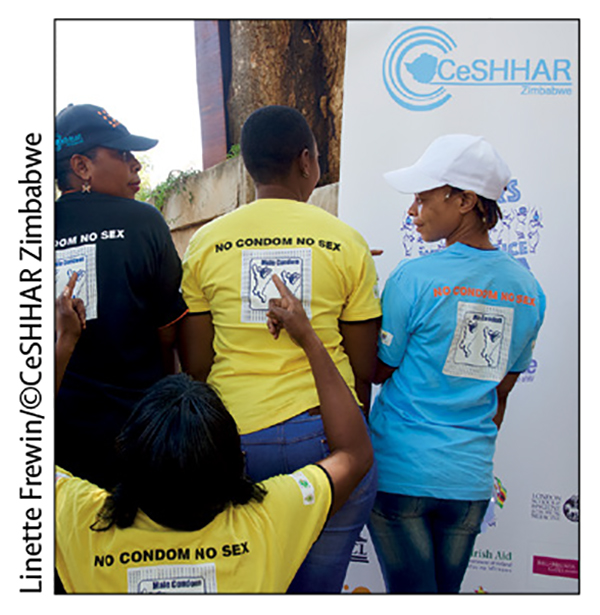

Prevention of HIV includes behavioural, biomedical, and structural approaches to reduce the risk of infection and transmission.^1^ Although randomised controlled trials have shown the efficacy of antiretroviral-based prevention interventions in isolation,^2,3^ their integration with behavioural and structural interventions might be required to have the maximum effect on the health of populations most affected by HIV.^4^ Tailored combination prevention activities could hold particular promise for substantially altering the trajectory of the HIV epidemic in historically disenfranchised communities, such as sex workers.^4,5^

Female sex workers are 13·5 times more likely to be infected with HIV than similarly aged women in the general population,^6^ yet they face increased barriers to access, uptake, and continuation of HIV prevention and care. The reasons are complex and multifaceted, and include stigma and discrimination, oppressive legal environments, concerns related to confidentiality, and fear of harassment and abuse.^7^ Generation of evidence supporting combination prevention approaches that address these barriers is crucial to develop the HIV response.

In *The Lancet HIV*, Frances Cowan and colleagues^8^ report the results of the SAPPH-IRe trial, a cluster-randomised trial in which a combination prevention intervention was designed to build on the existing national free sexual-health services already provided for female sex workers in Zimbabwe by Sisters with a Voice (Sisters). This programme provides peer-educator-supported sexual and reproductive health services, referral for HIV care, peer support, and legal advice, and was used in the usual-care group. The prevention intervention added community engagement to increase awareness of antiretroviral-based treatment and prevention, strengthen support networks, reduce barriers to starting antiretroviral therapy (ART) or pre-exposure prophylaxis (PrEP), and facilitate adherence. The results are striking. 14 clusters were assessed, seven in the usual-care group (3612 women seen in clinics) and seven in the intervention group (4619 women). Among women living with HIV, the proportions with viral suppression (viral load <1000 copies per mL) were 72% in the intervention group and 68% in the usual-care group. Despite stable HIV prevalence over the study period, awareness of status, uptake of ART, and virological suppression increased in the two study groups, although by a similar degree in both. The quality of care in the usual-care group of the SAPPH-IRe trial was considerably higher than that available to many female sex workers living and working in sub-Saharan Africa, and might have empowered and supported women to access services and facilitated their engagement in HIV prevention and treatment, leading to the lack of difference between groups.

Despite the encouraging results, important opportunities for improving HIV prevention services for female sex workers remain. Although nearly 40% of HIV-uninfected participants in Cowan and colleagues’ intervention group started PrEP, average retention was only just over 4 months. When taken consistently, PrEP is highly effective at reducing the risk of HIV infection.^9^ As a user-controlled intervention, PrEP can be an empowering option for female sex workers who are unable to negotiate condom use. However, engagement in PrEP care requires frequent interaction with the health system.^10^ To maximise the potential of PrEP, therefore, combination prevention approaches will probably need to support long-term engagement. Such approaches could build on the Sisters model and include peer support and community-based empowerment activities for PrEP and incorporate HIV self-testing to reduce the overall number of visits to health facilities.^11,12^

To end the HIV epidemic, combination prevention approaches are likely to be necessary, and a one-size-fits-all approach will not suffice. The results of the SAPPH-IRe trial^8^ indicate that achieving high HIV testing coverage and viral suppression is possible with tailored interventions for female sex workers, but availability of culturally appropriate services varies in areas with high HIV prevalence. Women who engage in sex work are members of diverse and heterogeneous communities, and many do not identify as sex workers. Therefore, what is deemed acceptable, suitable, or both, might also vary substantially across places and populations. Evidence of the effects of tailored, innovative, and scalable interventions for specific populations, such as that assessed in the SAPPH-IRe trial, in diverse settings and populations is urgently needed to provide the best services to and reduce HIV transmission in the populations most in need.

## References

[1] Vermund SH, Hayes RJ. Combination prevention: new hope for stopping the epidemic. Curr HIV/AIDS Rep 2013; 10: 169–86. 23456730 10.1007/s11904-013-0155-y PMC3642362

[2] Baeten JM, Donnell D, Ndase P, Antiretroviral prophylaxis for HIV prevention in heterosexual men and women. N Engl J Med 2012; 367: 399–410. 22784037 10.1056/NEJMoa1108524 PMC3770474

[3] Cohen MS, Chen YQ, McCauley M, Antiretroviral therapy for the prevention of HIV-1 transmission. N Engl J Med 2016; 375: 830–39. 27424812 10.1056/NEJMoa1600693 PMC5049503

[4] Bekker L-G, Johnson L, Cowan F, Combination HIV prevention for female sex workers: what is the evidence? Lancet 2015; 385: 72–87. 25059942 10.1016/S0140-6736(14)60974-0 PMC10318470

[5] Beyrer C, Crago A-L, Bekker L-G, An action agenda for HIV and sex workers. Lancet 2015; 385: 287–301. 25059950 10.1016/S0140-6736(14)60933-8 PMC4302059

[6] Baral S, Beyrer C, Muessig K, Burden of HIV among female sex workers in low-income and middle-income countries: a systematic review and meta-analysis. Lancet Infect Dis 2012; 12: 538–49. 22424777 10.1016/S1473-3099(12)70066-X

[7] Shannon K, Strathdee SA, Goldenberg SM, Global epidemiology of HIV among female sex workers: influence of structural determinants. Lancet 2014; 385: 55–71. 25059947 10.1016/S0140-6736(14)60931-4 PMC4297548

[8] Cowan FM, Davey C, Fearon E, Targeted combination prevention to support female sex workers in Zimbabwe accessing and adhering to antiretrovirals for treatment and prevention of HIV (SAPPH-IRe): a cluster-randomised trial. Lancet HIV 2018; published online July 17. 10.1016/S2352-3018(18)30136-X. 30030134

[9] Cowan FM, Delany-Moretlwe S. Promise and pitfalls of pre-exposure prophylaxis for female sex workers. Curr Opin HIV AIDS 2016; 11: 27–34. 26633639 10.1097/COH.0000000000000215

[10] Nunn AS, Brinkley-Rubinstein L, Oldenburg CE, Defining the HIV pre-exposure prophylaxis care continuum. AIDS 2017; 31: 731–34. 28060019 10.1097/QAD.0000000000001385 PMC5333727

[11] Chanda MM, Ortblad KF, Mwale M, HIV self-testing among female sex workers in Zambia: A cluster randomized controlled trial. PLoS Med 2017; 14: e1002442. 29161260 10.1371/journal.pmed.1002442 PMC5697803

[12] Ortblad K, Kibuuka Musoke D, Ngabirano T, Direct provision versus facility collection of HIV self-tests among female sex workers in Uganda: a cluster-randomized controlled health systems trial. PLoS Med 2017; 14: e1002458. 29182634 10.1371/journal.pmed.1002458 PMC5705079

